# Differential Interactions of Sex Pheromone and Plant Odour in the Olfactory Pathway of a Male Moth

**DOI:** 10.1371/journal.pone.0033159

**Published:** 2012-03-12

**Authors:** Nina Deisig, Jan Kropf, Simon Vitecek, Delphine Pevergne, Angela Rouyar, Jean-Christophe Sandoz, Philippe Lucas, Christophe Gadenne, Sylvia Anton, Romina Barrozo

**Affiliations:** 1 UMR 1272 Physiologie de l'Insecte: Signalisation et Communication, INRA, Route de Saint-Cyr, Versailles, France, Université Pierre et Marie Curie, 7 Quai Saint Bernard, Paris, France; 2 CNRS, UMR 5169, Université Paul Sabatier, Research Center for Animal Cognition, Toulouse, France; Center for Genomic Regulation, Spain

## Abstract

Most animals rely on olfaction to find sexual partners, food or a habitat. The olfactory system faces the challenge of extracting meaningful information from a noisy odorous environment. In most moth species, males respond to sex pheromone emitted by females in an environment with abundant plant volatiles. Plant odours could either facilitate the localization of females (females calling on host plants), mask the female pheromone or they could be neutral without any effect on the pheromone. Here we studied how mixtures of a behaviourally-attractive floral odour, heptanal, and the sex pheromone are encoded at different levels of the olfactory pathway in males of the noctuid moth *Agrotis ipsilon*. In addition, we asked how interactions between the two odorants change as a function of the males' mating status. We investigated mixture detection in both the pheromone-specific and in the general odorant pathway. We used a) recordings from individual sensilla to study responses of olfactory receptor neurons, b) *in vivo* calcium imaging with a bath-applied dye to characterize the global input response in the primary olfactory centre, the antennal lobe and c) intracellular recordings of antennal lobe output neurons, projection neurons, in virgin and newly-mated males. Our results show that heptanal reduces pheromone sensitivity at the peripheral and central olfactory level independently of the mating status. Contrarily, heptanal-responding olfactory receptor neurons are not influenced by pheromone in a mixture, although some post-mating modulation occurs at the input of the sexually isomorphic ordinary glomeruli, where general odours are processed within the antennal lobe. The results are discussed in the context of mate localization.

## Introduction

Most animals rely on olfactory cues to find their mating partner, food and shelter. For reproduction, the olfactory system faces the challenge of extracting salient odorant information emitted by sexual partners (pheromones) from an abundant background of general odorants. In the moth's natural environment, males are attracted by a female-emitted sex pheromone blend (containing several components), and could either ignore or use background general odours as additional cues to locate a potential mate. Indeed, in several moth species, the behavioural response of males to sex pheromones is enhanced by host plant odours [1]. This seems to reflect a strategy to optimize mating, since females often call when situated on a host plant. The simultaneous presence of a pheromone and a plant odour may result in interactions between these odour classes, which can either lead to suppression (masking) or enhancing (synergy) of the response to one odour by the other.

Detection of sex pheromones and general odours in animals is usually accomplished by two distinct olfactory pathways. In mammals, pheromone information is mainly processed by the accessory olfactory system, while the main olfactory system codes more general odours, *e.g.* food or shelter related odours [2]. In insects, such as moths, pheromone information is transmitted by specialized olfactory receptor neurons (ORNs) to the macroglomerular complex (MGC), a male-specific part of the primary olfactory processing centre, the antennal lobe (AL). Plant odour information is transferred by general ORNs to sexually isomorphic ordinary glomeruli (OG) [3]. Whereas in mammals both sub-systems seem to participate in mate recognition [2], very little is known about how both sub-systems contribute to pheromone and plant odour recognition in moths.

Pheromone-plant odour interactions may occur at different processing levels in the olfactory system. Olfactory mixture perception has already been studied at the peripheral level (vertebrates: *e.g.* [4], [5]; invertebrates: *e.g.* [6]–[11]) and at the central level (vertebrates: *e.g.* [12], [13]; invertebrates: *e.g.* [14]–[18]). However, most of these studies investigated coding of mixtures composed of odorants from the same contextual origin (*i.e.* mixtures of either general odorants or single pheromone components). Very few studies have focussed on the coding of mixtures of pheromones (reproduction cues) and general odours (*i.e.* food, predator, social, host cues) in the central nervous system (e.g. vertebrates [19], [20], and invertebrates [21], [22]). As for vertebrates, the coding of these two types of odour cues was generally believed to occur in two separate pathways of the insect olfactory system. However, unusual representations of plant odours and pheromones were recently observed in tortricid moths: in *Grapholita molesta*, pheromone processing seems to occur in OG rather than in the MGC [23] and in *Cydia pomonella* there is no clear segregation between the pheromone and the general odour sub-systems in the AL, both odour classes being represented in both the MGC and in OG [24].

In males of the noctuid moth *Agrotis ipsilon*, a transient post-mating inhibition of behavioural and central nervous responses to sex pheromone has been observed [21], [25]. This plasticity prevents newly-mated males from orientating towards females and mating until the next night, allowing them to refill their sex glands for a potential new ejaculate. After mating, a strong decrease in sex pheromone sensitivity is observed up to the MGC [26]. Plant-odour processing, on the other hand, is much less affected by mating status. Behavioural responses to plant odours, such as a linden flower extract, observed in wind tunnel experiments remain stable after mating. Further, response thresholds of peripheral and central OG neurons to heptanal, a behaviourally attractive component of linden flower [27], are not modified [21], [26]. However, an increase in calcium response intensity and in the firing response of OG neurons to heptanal is observed, originating probably from pre-synaptic modulation at ORN axon terminals [26]. Thus, pheromone and plant odour processing seem to be modulated differentially depending on the mating status. This plasticity allows mated males to transiently block their central pheromone responses after mating and to increase non-pheromonal odour detection, probably allowing more efficient localization of food sources in a natural environment [26].

Interestingly, the addition of plant odour (linden flower extract) enhanced the response of virgin males to sex pheromone, which in turn inhibited the response of mated males to plant odour both at the behavioural and central nervous level (within OG) [21].

Here we used a multi-level approach to investigate pheromone-plant odour interactions in the olfactory pathway of virgin and mated *A. ipsilon* males. In a first step, we tested the behavioural response of virgin males to heptanal using wind tunnel experiments to confirm previous reports of its attractiveness in the field also in the laboratory [27]. We analysed responses to a sex pheromone blend/plant odour (heptanal) mixture a) in pheromone-specific and heptanal-responding ORNs; b) globally in the AL by recording the calcium signal elicited within the two sub-systems of the AL, the MGC and the OG; and c) in projection neurons (PNs), branching in the MGC and leaving the AL towards higher order brain centres. We compared these data with previously described odour interactions within OG glomeruli [21]. The plant odour heptanal strongly suppressed the response of pheromone-specific ORNs (Phe-ORNs) to the sex pheromone blend. This effect was confirmed for all the different levels of the pheromone-specific olfactory pathway we investigated, independently of the mating status. Conversely, there was no modulation of heptanal-sensitive receptor neurons (Hep-ORNs) by the pheromone when presented together in a mixture with heptanal. In addition, mated males showed higher response intensities to heptanal and mixtures in calcium imaging recordings from OG than virgin males.

## Results

### Behavioural responses of virgin males to heptanal

**Figure 1:**
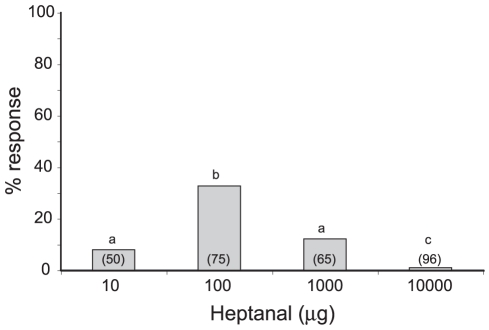
Behavioural responses of virgin *A. ipsilon* males to heptanal. The proportion of males showing an oriented flight towards the stimulus source was highest at a dose of 100 µg heptanal. Numbers in brackets represent the numbers of tested males. Bars with same letters are not statistically different (chi-square-test, p<0.05).

To confirm the behavioural attractiveness of heptanal, a volatile emitted by linden flowers, we performed wind tunnel experiments using virgin sexually mature *A. ipsilon* males. Best responses (32% males responding with an oriented flight) were obtained with 100 µg heptanal (Figure 1).

### Pheromone-ORN responses are reduced by heptanal

**Figure 2:**
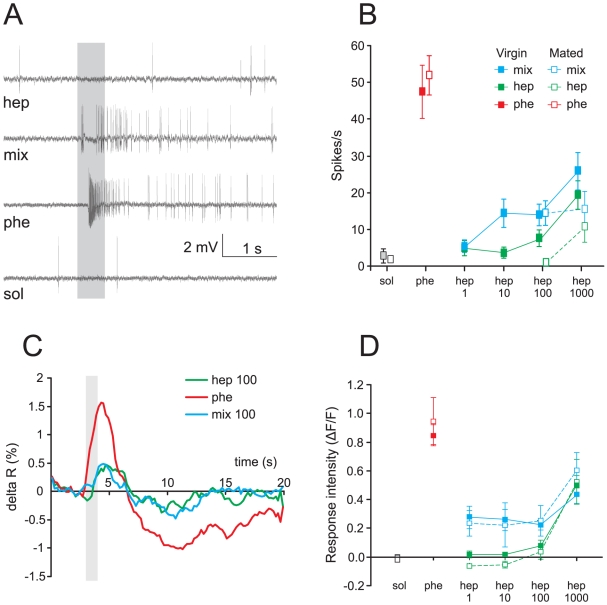
Pheromone-responding ORNs and MGC calcium responses in virgin and mated males. **A**) Typical single sensillum recordings showing an olfactory receptor neuron (ORN) excitatory response to the pheromone (10 ng), no response to heptanal (100 µg) and a reduced response to the pheromone/heptanal mixture in a virgin male. Solvent = hexane. The grey bar indicates the duration of the stimulus (0.5 s). **B**) Mean spike frequency of Phe-ORNs to the sex pheromone (10 ng), heptanal at different doses, and their mixture in virgin (n = 14) and mated (n = 22) males. Solvent (sol) refers to pooled data of stimulations with hexane and mineral oil. Phe-ORNs show a decreased firing frequency to the pheromone by the addition of heptanal at all doses tested. Phe-ORNs do not respond to heptanal as single odour except for the highest dose tested (1000 µg). No differences were found between virgin and mated males. **C**) Time course of odour-evoked calcium activity in the MGC of one mated male. The grey bar indicates the duration of the stimulus (1 s). **D**) Mean calcium responses in the MGC of virgin (n = 9) and mated (n = 8) males to sex pheromone (10 ng), different doses of heptanal and their mixtures. Stimulation with heptanal in the mixture strongly reduced the response intensity to pheromone at any dose of heptanal tested. Heptanal only induced calcium response for the highest dose tested (1000 µg). No differences were found between virgin and mated males. Hep: heptanal; mix: pheromone/heptanal mixture; phe: pheromone; sol: solvent.

Phe-ORNs housed in long trichoid sensilla on the antennae showed a typical excitatory response to the sex pheromone blend, but no response to heptanal or to the solvent. Spiking activity to a mixture of pheromone and heptanal was strongly reduced compared to the response evoked by the pheromone alone (Figure 2A).

We first analysed the effect of the mating status on the response of Phe-ORNs to pheromone, heptanal and their mixture at two doses (100 and 1000 µg) (Figure 2B). Phe-ORNs of virgin and mated insects displayed no significant differences in the spike response frequencies to the stimuli tested (3-way RM ANOVA, mating factor: F_1,29_ = 0.25, p = 0.62) (Figure 2B), but significant differences were found with respect to the other two factors: odour and doses tested (3-way RM ANOVA, odour factor: F_1,29_ = 100.3, p = 0.00001; dose factor: F_1.6,45.9_ = 21.9, p = 0.00001). As the mating status of males did not interplay in the differences observed, we further analysed only data from virgin males. Similar statistical differences as before were detected by performing a 2-way RM ANOVA with odours and doses as the two main factors (RM factors) (2-way RM ANOVA, odour factor: F_1,13_ = 48.5, p = 0.00001; dose factor: F_1.8,23.3_ = 9.5, p = 0.001) (Figure 2B). Addition of heptanal to the pheromone significantly reduced the firing rate of Phe-ORNs at all tested doses (simple effects, 1-way RM ANOVA F_1.6,20.5_ = 14.9, p = 0.00001, Tukey test, p<0.005 in all cases, i.e. 1–1000 µg of mix *vs.* phe). Heptanal doses between 1 and 100 µg (Figure 2B, green) evoked no significant responses with respect to the solvent (simple effects, 1-way RM ANOVA: F_1.4,18.9_ = 4, p = 0.04, Tukey test: 1–100 µg of hept vs. sol, p>0.9 in all comparisons). However, a high dose of 1000 µg of heptanal elicited a significant response in Phe-ORNs as compared to the solvent (Tukey test: 1000 µg of hep *vs.* sol, p = 0.006). Thus, pheromone responses of Phe-ORNs were reduced by addition of heptanal at doses, irrespectively if they elicited or not responses on their own (which was the case only for the highest dose).

### Pheromone-evoked calcium responses in the MGC are reduced by heptanal

**Figure 3:**
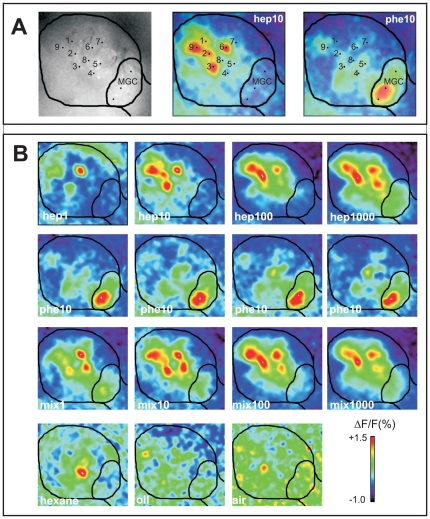
Odour-evoked calcium signals in the antennal lobe. **A**) Example of an anatomical staining of a right antennal lobe (AL) with the outline of the entire AL and MGC. Two activity maps obtained in response to heptanal (10 µg) (hep10) and to the pheromone blend (10 ng) (phe10) are shown with the outline of the AL. Numbers next to dots indicate the position of the nine analysed ordinary glomeruli (1–9), as well as three analysed locations within the MGC, for which activity was pooled. **B**) Activity signals obtained in a mated male stimulated with four doses of heptanal (hep) (1–1000 µg), four presentations of pheromone (phe) at 10 ng, and the respective pheromone/heptanal mixtures (mix). All maps are scaled to the same minimum/maximum.

Odour–evoked calcium responses were typical biphasic signals with a fast fluorescence increase followed by a slow decrease before returning to baseline (Figure 2C). The maximum intensity of the response to odour stimuli appeared around 1 s after stimulation onset, whereas the minimum was found around 5 s after odour onset. Controls (hexane, mineral oil, clean air) did not activate the AL (Figure 3B).

The sex pheromone induced high calcium responses in the MGC, and no responses to heptanal alone were observed, apart for the highest heptanal dose (Figure 2D, 3A, B). MGC calcium responses did not vary with mating status (3-way RM ANOVA, mating factor: F_1,15_ = 0.0026, p = 0.96), but did with odour (3-way RM ANOVA, odour factor: F_1,15_ = 43.4, p = 0.000009) and with the heptanal dose both as single odour or in the mixture (3-way RM ANOVA, dose factor: F_2.3,34.6_ = 28.3, p = 0.0000001) (Figure 2D). As for electrophysiological responses of Phe-ORNs, the addition of heptanal to the pheromone, at any of the heptanal doses tested, strongly reduced MGC response intensity (simple effects, 1-way RM ANOVA: F_2.7,44.4_ = 29.4, p = 0.000001, Tukey test for phe vs. 1–1000 µg of mix, p<0.0001 in all cases) (Figure 2D). Similarly to single sensillum recordings, we only observed a calcium response to heptanal in the MGC for the highest dose (1000 µg) (simple effects, 1-way RM ANOVA: F_1.4,23.2_ = 35.5, p = 0.000001, Tukey test, 1–100 µg hep *vs.* sol, p>0.7; 1000 µg *vs.* sol or any other hep dose, p<0.0001 in all cases) (Figure 2D). Concluding, as in Phe-ORN recordings, pheromone-induced responses in the MGC were reduced by addition of heptanal at doses, which did not elicit responses on their own, with the exception of the highest dose.

### Heptanal-ORN responses are not affected by pheromone

**Figure 4:**
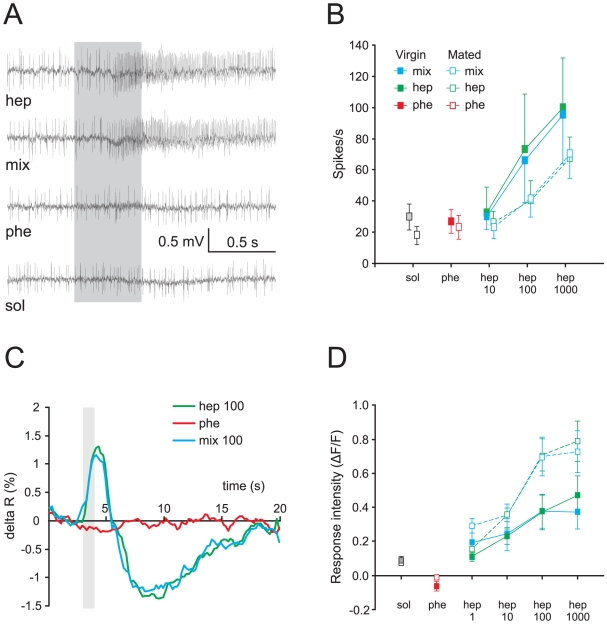
Heptanal-sensitive ORNs and OG calcium-evoked responses in virgin and mated males. **A**) Typical recording showing an excitatory response to heptanal (100 µg), no response to the pheromone (10 ng) and the solvent (mineral oil), and excitation to the pheromone/heptanal mixture in a virgin male. The grey bar indicates the duration of the stimulus (0.5 s). **B**) Mean spike frequency of Hep-ORNs to pheromone (10 ng), heptanal at different doses, and their mixture in virgin (n = 13) and mated (n = 13) males. Hep-ORNs show dose-dependent response to heptanal, but no response to the pheromone and solvent. The addition of pheromone in the mixture does not modify the response of Hep-ORNs to heptanal at any dose tested. No differences were detected between virgin and mated males. **C**) Time course of odour-evoked calcium activity in the OG. The grey bar indicates the duration of the stimulus (1 s). **D**) Mean calcium responses in the OG to pheromone (10 ng), heptanal at different doses, and their mixture in virgin (n = 9) and mated (n = 8) males. Stimulation with pheromone induced no response. Heptanal-induced responses increased with the dose and were significantly higher in mated than in virgin males, although it was not different from mixture responses. Hep: heptanal; mix: pheromone/heptanal mixture; phe: pheromone; sol: solvent.

Extracellular recordings from individual Hep-ORNs housed in short sensilla trichodea on the antennae revealed excitatory responses to heptanal and to the pheromone/heptanal mixture (Figure 4A). No responses were detected when the pheromone blend alone or the solvent were presented (Figure 4A). The spiking rate of Hep-ORNs was not significantly different between virgin and mated males (3-way RM ANOVA, mating factor: F_1,24_ = 0.63, p = 0.43). Moreover, no difference in Hep-ORN firing rate was observed between responses to heptanal and to the pheromone/heptanal mixture (3-way RM ANOVA, odour factor: F_1,24_ = 0.18, p = 0.67) (Figure 4A, B). However, the Hep-ORN spike frequency significantly increased with the dose of heptanal in both groups (3-way RM ANOVA, dose factor: F_1.2,29.9_ = 14.2, p = 0.00032) (Figure 4B). Thus, Hep-ORNs responded in a dose dependent manner to heptanal, and their responses were neither affected by the addition of pheromone, nor by a change in mating status.

### Heptanal-evoked calcium responses in OG are not affected by sex pheromone

OG calcium responses to heptanal or to the pheromone/heptanal mixture were biphasic signals and their time course was comparable to responses observed in the MGC (Figure 2C, 4C). In the OG, no calcium responses to sex pheromone blend stimulation were detected (Figure 3A, B and 4C).

Across all tested stimuli and doses, response intensities were significantly different between virgin and mated males (3-way RM ANOVA, mating factor, F_1,15_ = 4.6, p = 0.048) and dose-dependent in both groups (3-way RM ANOVA, dose factor, F_1.9,29.2_ = 39.7, p = 0.0000001) (Figure 4D). Mated males showed higher responses particularly for doses of 100 and 1000 µg of heptanal alone and for the corresponding pheromone/heptanal mixtures than virgin males (simple effects, 1-way ANOVA: F_1,32_ = 10.8, p = 0.002 for 100 µg and F_1,32_ = 9.2, p = 0.004 for 1000 µg) (Figure 4D). The analysis revealed no significant differences in OG response intensity between heptanal and the pheromone/heptanal mixture in both groups (3-way RM ANOVA, odour factor: F_1,15_ = 0.49, p = 0.49) (Figure 4D). Thus dose-dependent calcium responses to heptanal in OG were not modified by the addition of sex pheromone and were higher in mated than in virgin males.

### Pheromone responses in MGC PN neurons are reduced by heptanal

**Figure 5:**
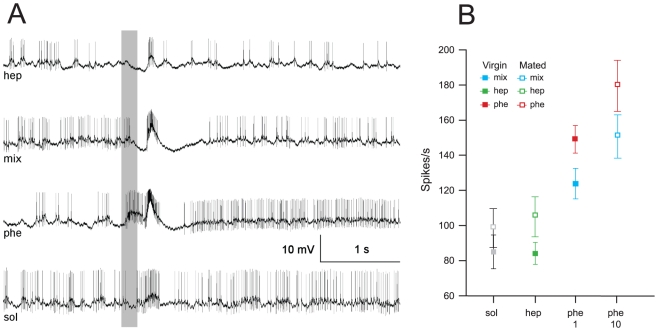
Responses of AL PNs within the MGC of virgin and mated males. **A**) Typical responses of a pheromone-sensitive PN in a virgin male, showing an excitatory response to pheromone (1 ng), no response to heptanal (100 µg) and the solvent (hexane), and a reduced firing rate during excitation to the pheromone/heptanal mixture. The grey bar indicates the duration of the stimulus (0.2 s). **B**) Spike frequency of PNs during the excitatory period to the pheromone (1 ng in virgin and 10 ng in mated males), heptanal (100 µg) and the pheromone/heptanal mixture in virgin (n = 17 neurons) and mated (n = 15 neurons) males. Spike frequencies of PNs do not differ between heptanal and solvent. Hep: heptanal; mix: pheromone/heptanal mixture; phe: pheromone; sol: solvent.

We describe here the analysis of the most common type of response pattern (97% of the recorded neurons) to the sex pheromone blend in MGC PN neurons, consisting of an excitatory followed by an inhibitory phase [28]. MGC PN response thresholds are lower in virgin than in mated males (*e.g.* [21]). We thus stimulated virgin males with a lower dose of pheromone (1 ng instead of 10 ng used in all other experiments) to avoid too strong responses. MGC PN responses were significantly different between the odours tested, *i.e.* solvent, heptanal, pheromone and pheromone/heptanal mixture (1-way RM ANOVA: F_3,45_ = 49.9, p = 0.00001 for virgin males and F_3,33_ = 24.8, p = 0.00001 for mated males) (Figure 5B). MGC PNs did not respond to heptanal (Figure 5A, B) as spike frequency did not differ between heptanal and solvent presentation, independently of mating state (Tukey test, hep vs sol: p = 0.99 for virgin males and p = 0.8 for mated males). In virgin males, addition of heptanal to 1 ng of the sex pheromone blend caused a reduction of the response (Tukey test, phe *vs.* mix, p = 0.001) (Figure 5B). The same type of effect was found in mated males when heptanal was added to 10 ng of the pheromone (Tukey test, phe vs. mix, p = 0.04) (Figure 5B). Thus, at the level of MGC PNs, addition of heptanal reduces the response to the pheromone. This effect was observed independently of the mating status. The effect of mating on the sensitivity of PNs could, however, not be directly compared statistically here, as different doses of pheromone were used for virgin and mated males.

## Discussion

Using complementary methodological approaches, our study examines the coding of a sex pheromone/heptanal mixture at different levels of the olfactory pathway in an insect. In addition, we evaluated the effect of mating status on the processing of such a mixture in mature *A. ipsilon* males. We show that mixtures composed of the pheromone and the plant odour heptanal are differentially detected and processed by the olfactory pathway: heptanal strongly suppresses pheromone detection, but conversely the pheromone does not affect heptanal detection. The pheromone response suppression caused by heptanal starts at the periphery (the antennae) and persists throughout the output of the pheromone-specific part of the AL, the MGC. Heptanal detection, on the other hand, does not seem to be modulated by the pheromone, at least up to the input to the OG. Mating status did not affect mixture processing in the pheromone-specific antennal and AL pathway (Phe-ORN, input and output of the MGC), but induced a plasticity of responses to heptanal and to the pheromone/heptanal mixture at the input to the OG. Further, we confirmed that heptanal, which was used as a plant odour throughout this study, is behaviourally attractive to *A. ipsilon* males in the wind tunnel, the level of response being similar to that obtained after stimulation with a linden flower extract [21].

### Pheromone-plant odour interactions at the peripheral level

Our results show that addition of heptanal strongly inhibits the responses of Phe-ORNs to the sex pheromone. Neither inhibitory nor excitatory responses (unless very high doses were used) were obtained in these neurons upon heptanal stimulation alone. Different forms of interactions have been found at the peripheral level after stimulation with mixtures of plant odours and pheromone. In *Helicoverpa zea*, linalool and a green leaf volatile were found to increase the response of Phe-ORNs when presented simultaneously with the main pheromone component [11]. On the contrary, a decrease of Phe-ORN response induced by the addition of plant odours has been found in various moth species such as *Antheraea pernyi* [29], *Adoxophyes orana* [30], *Bombyx mori* [6], and *Spodoptera littoralis* [31]. The suppressive effect of plant odours on Phe-ORN responses, as observed in our and the above cited studies, might originate from non-competitive inhibition of pheromone and plant volatile compounds for olfactory receptors or other actors involved in signal reception, or could be due to an inhibition of the transduction pathway as proposed for *B. mori* [32]. Our results show that the lowest dose of heptanal used (1 µg) was enough to reduce the firing rate of Phe-ORNs to the pheromone. Further experiments are necessary to determine the threshold dose of heptanal needed to produce this suppression effect. Even 1000 µg of heptanal in the mixture evoked a significant reduction of the pheromone response in spite of the fact that when presented alone, it elicited significant firing rates in Phe-ORNs. In contrast to the suppressive effect of heptanal on pheromone responses in Phe-ORNs, Hep-ORNs not only did not respond to the pheromone alone but their responses were not modified by the presence of sex pheromone in the mixture. This suggests the existence of different peripheral interactions between plant odour and pheromones depending on the ORN type.

### Pheromone-plant odour interactions at the AL level

**Figure 6:**
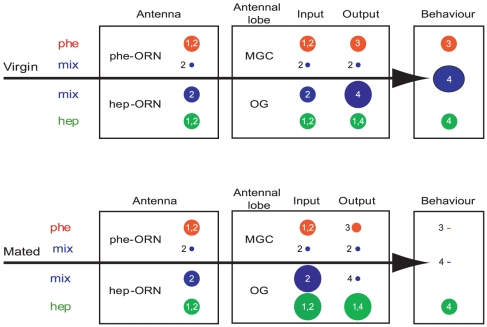
Sex pheromone-plant odour interactions in the olfactory pathway of virgin and mated *A. ipsilon* males. Whereas pheromone sensitivity decreases drastically in AL output neurons after mating, heptanal sensitivity seems to increase already at the AL input level. Synergistic behavioural responses to odour mixtures in virgin males are correlated with enhanced antennal lobe responses. Likewise inhibitory behavioural responses to mixtures of pheromone and plant odour in mated males match inhibitory interactions within ordinary glomeruli of the antennal lobe. Pheromone reception and antennal lobe processing, on the other hand are inhibited by heptanal, independently of mating state. This might serve to improve temporal resolution of discontinuous stimuli, which are common in a natural environment. AL: antennal lobe; hep: heptanal; MGC: macroglomerular complex; mix: heptanal/pheromone mixture; OG: ordinary glomeruli; ORN: olfactory receptor neuron; phe: pheromone. Size of disks indicates response strength. Dash means no response. Numbers refer to previously published data: (1) Barrozo et al., 2011 [26] (2) This paper. (3) Gadenne et al., 2001 [25]. (4) Barrozo et al., 2010 [21].

Our calcium imaging experiments revealed a compound signal response consisting mainly of ORN responses [33], [34]. Calcium responses measured at the level of the MGC were consistent with those obtained from single sensillum recordings of Phe-ORNs (Figure 6). Thus, the suppressive interactions observed within the MGC largely originate from interactions at the peripheral level. In addition, as shown by our PN recordings, this effect does not seem to be modified within the AL, *i.e.* input = output (Figure 6). Interestingly, the type of interactions we found between sex pheromone and heptanal in *A. ipsilon* is different from that found in the silkmoth *B. mori*, in which sex pheromone responses in PNs of the MGC are enhanced by the host plant odour cis-3-hexen-1-ol [22]. There might thus either be different effects of different plant volatiles on sex pheromone detection, or different interaction effects in different moth species, depending on the natural context.

In contrast to the pheromonal pathway, we found no mixture interactions in the heptanal pathway, at least at its input level. Indeed, responses to heptanal were not affected by the addition of pheromone, neither in Hep-ORN recordings nor in odour-evoked calcium responses of OG. However it should be noted that in virgin *A. ipsilon*, a synergistic effect of mixtures of sex pheromone and heptanal has been demonstrated in OG output neurons, in correlation with a synergistic behavioural effect (Figure 6) [21]. Contrary to interactions in the pheromonal pathway, which appear at the periphery, interactions in the heptanal pathway would be the product of AL processing. Although the neural mechanisms leading to mixture interactions within the AL still need to be unveiled, they may mainly originate from lateral interactions through inhibitory or excitatory local interneurons [35], [36].

### Odour interactions as a function of mating state

We did not find any differences in mixture interactions at the input or output of the AL pheromone pathway and in Hep-ORNs between virgin and mated males. However, OG calcium responses (this paper) and heptanal-responding OG PNs [21] were modulated by mating status. OG PNs of virgin males showed enhanced responses to the mixture compared to heptanal alone, while mated males exhibited reduced responses to the mixture compared to heptanal alone (Figure 6) [21]. These mixture interactions observed in OG PNs were correlated with the behavioural synergism or inhibition to mixtures observed in virgin and newly-mated males, respectively (Figure 6) [21]. It is important to state here that the reduction of pheromone detection by plant odours has been described in several moth species [6], [29], [30], [32]. In recent studies on the peripheral and central olfactory system, this inhibition has been shown to improve pulse resolution of pheromone stimuli [31], [37], [38] an important feature to allow orientation towards a naturally intermittent pheromone signal [39].

Although reciprocal modulation of heptanal and sex pheromone processing is clear in our model insect, our results show that interaction mechanisms occur at different levels in moths. Whereas the modulation of pheromone responses by heptanal is essentially happening in the periphery, probably due to competition for the olfactory receptors, considerable mating-dependent plasticity and signal processing of mixtures occurs within the OG at the AL level (Figure 6).

### Conclusions

The ecological importance of the co-occurrence of different classes of odours involved in different behavioural contexts is evident in the natural environment of an insect. The present study is a first step to better understand how a male moth processes crucial information cues for reproduction (sex pheromone) in a complex odorous environment (plant odours), and how the reproductive state might modulate its response. Our data, showing reciprocal modulation of the two types of stimuli, give some indications about how a male moth can process cues originating simultaneously from a mating partner and a plant odour background, ultimately leading to an appropriate behavioural response.

## Materials and Methods

### Insects

Adult males and females of the noctuid moth, *A. ipsilon* Hufnagel, were reared in the laboratory, and behavioural and physiological experiments were performed as described previously [21], [25]. Briefly, 5-day old sexually mature virgin and mated males were used for experiments during the 8-hour scotophase. Newly-mated males were obtained by pairing virgin 5-day-old males and 3-day-old sexually mature females before the onset of the scotophase. Newly–mated males were prepared for calcium imaging or electrophysiological recordings within one to two hours after the end of copulation, and females were dissected to confirm the presence of the male spermatophore.

### Odour Stimulation

For electrophysiological experiments, odour stimulations with sex pheromone blend and heptanal were performed as described previously [21], [40]. Briefly, the behaviourally active pheromone blend consisting of (*Z*)7-dodecen-1-yl acetate, (*Z*)9-tetradecen-1-yl acetate and (*Z*) 11-hexadecen-1-yl acetate at a ratio 4∶1∶4 [41], and the behaviourally attractive plant odour, heptanal [27], were used in all experiments. Ten ng of the pheromone blend diluted in hexane were used in ORN recordings and imaging experiments because they elicit a clear response [25]. For AL intracellular recordings 1 ng and 10 ng doses of the pheromone were used for virgin and mated males respectively, because of a higher sensitivity of central neurons in virgin males [21]. Four doses of heptanal (1, 10, 100 and 1000 µg each diluted in 10 µl of mineral oil, resulting in concentrations of 1/10.000 to 1/10 volume/volume) were used for electrophysiological recordings and imaging experiments. All compounds were purchased from Sigma Aldrich (Saint-Quentin Fallavier, France) and 10 µl of stimulus solution were applied on a piece of filter paper (0.5×2 cm, Fisherbrand, Fisher Bioblock, Illkirch, France) introduced in a Pasteur pipette. The solvents hexane and mineral oil applied on a filter paper were used as control stimuli. When stimulating with mixtures, two filter papers were inserted into a glass pipette; then the pheromone (diluted in hexane) and heptanal (diluted in mineral oil) were added separately on the filter papers. This procedure avoided interactions between the two odour solutions, but allowed simultaneous application of the two stimuli in the same air puff. To exclude potential absorption of the pheromone in mineral oil or changes in the airflow due to a second filter paper in the pipette, we carried out control experiments under exactly the same experimental conditions as in the main experiments, in which we stimulated Phe-ORNs with: 1- a single filter paper with pheromone (phe), 2- one filter paper with pheromone and a second clean filter paper (phe+cfp), 3- one filter paper with pheromone and a second filter paper with mineral oil (phe+oil), 4- a single filter paper with mineral oil (oil). Phe-ORN spike response frequencies did not change significantly when a second clean filter paper or a filter paper with mineral oil was inserted in the pipette with respect to the pheromone alone (Tukey test p>0.05), but as expected, responses were significantly lower when only mineral oil was used (Tukey test, oil vs. phe, phe+cfp, phe+oil, p<0.0001 in all cases) (1-way ANOVA for RM F_3,24_ = 60.3, p = 0.00001) (Fig. S1). Pipettes were left under a fumehood for 30 min to allow evaporation of hexane before use. Antennal stimulation was done with a stimulus controller (CS55, Syntech, Kirchzarten, Germany) as described before [40]. Stimulation lasted for 0.5 s for ORN recordings, 1 s for calcium imaging recordings and 0.2 s for PN recordings.

### Single sensillum recordings of ORNs

Preparation of animals and recordings were performed as described earlier [26], [40]. Briefly, recordings from pheromone sensilla were carried out according to the tip recording technique [42]. Recordings from plant odour sensilla were carried out with electrolytically sharpened tungsten wires. Sensilla were selected randomly along the stem in the middle part of the antenna. Responses for both sensillum types were calculated as the frequency of APs during the last 0.3 s period of the stimulation time (0.5 s) and mean responses and standard deviation were calculated for each stimulus in virgin and mated males.

### Calcium imaging

Animals were mounted individually in Plexiglas chambers and the head was fixed. The brain capsule was opened, glands and trachea removed, and then 20 µl dye solution (50 µg Calcium Green 2-AM dissolved with 50 µl Pluronic F-127, 20% in dimethylsulfoxide, Molecular Probes, Eugene, OR, USA) was bath-applied for a minimum of 1 hour, before being washed with Ringer. For recordings, a T.I.L.L. Photonics imaging system (Martinsried, Germany) was coupled to an epifluorescent microscope (Olympus BX-51WI, Olympus, Hamburg, Germany) equipped with a 10× (NA 0.3) water immersion objective. Signals were recorded using a 640×480 pixel 12-bit monochrome CCD camera (T.I.L.L. Imago, cooled to −12°C). Each animal was subjected to up to three series of olfactory stimulations with interstimulus intervals of 80 s. Identification of individual glomeruli was done by superposing activity maps using Adobe Photoshop (Version CS2). Raw data analysis was done using custom–made software written in IDL (Research Systems Inc., Colorado, USA) and Visual Basic (Microsoft Excel) according to previous work [8]. Briefly, after noise filtering and bleaching correction, relative fluorescence changes (ΔF/F) were calculated as (F−F_0_)/F_0_ (F_0_ = reference background). For each glomerulus, the time course of ΔF/F was calculated by averaging 25 pixels (5×5) at the centre of each glomerulus. Nine ordinary glomeruli (OG) were identified in all preparations (named 1–9) and average signals from the 9 glomeruli were calculated for each stimulus. For the MGC, due to its important size three locations were analysed and their data pooled, as they were not significantly different (Figure 3A).

### Intracellular recordings of AL neurons

Preparation, intracellular recordings and response analysis of AL neurons from virgin and newly-mated males were performed as described previously [21]. PNs were randomly impaled within the array of the MGC. Data were recorded and analysed using Autospike 32 software (Syntech, The Netherlands). Numbers of APs elicited by a stimulus during the excitatory phase of the response (starting 0.2 s after the onset of stimulation and lasting about 0.4 s) were determined and mean response frequencies and standard deviations for PNs were calculated for each stimulus for virgin and mated males.

### Behavioural tests

Behavioural tests were performed in a wind tunnel as described previously [21]. Briefly, 5-day-old mature virgin males were exposed during mid-scotophase in a 2 m-long wind tunnel and their response was quantified to evaluate attractiveness of the stimulus. Males were transferred before the onset of scotophase from their rearing chamber into the wind tunnel room. For stimulation, heptanal diluted in mineral oil was used at four doses (10; 100; 1000; 10000 µg in 10 µl). Stimuli were dispensed on a filter paper and placed in the airflow upwind to the release site in the wind tunnel on a vertical holder. Each experimental male was tested only once to one stimulus and at a single dose, and then the animal was discarded. Assays were performed during 3 min, and partial flight, complete flight and landing on the pheromone source were considered as an oriented response [43], [44]. The proportion of males performing an oriented flight was analysed.

### Statistical Analysis

For electrophysiological experiments, data were analysed using 3-way repeated measures (RM) ANOVAs including 3 main factors: one fixed factor for mating status, and two RM factors for odour and dose. A 2-way RM ANOVA was carried out by excluding the mating factor of the analysis but keeping the RM factors odour and dose. Odours and doses were always considered as RM since they were presented one after the other in the same preparation. For the analysis, the pheromone as single odour was considered as the first point of the curve of mixtures, as it corresponds to a case of mixture without heptanal (*i.e.* phe+hep 0 µg). Responses to hexane and mineral oil were pooled and considered one data point: ‘solvent’. Further, solvent was included as the first point in the curve of heptanal (*i.e.* hep 0 µg) (Figs. 2B, D and 4B, D). When interactions among factors were significant, the simple effects were analysed by means of 1-way ANOVA with or without the RM factor, and then followed by Tukey test for post-hoc comparisons if necessary.

Virgin and mated males were analysed separately in Figure 5B by means of a 1-way RM ANOVA (main factor: odour, with four levels: solvent, heptanal, pheromone and the pheromone/heptanal mixture). In this experiment, virgin and mated males were stimulated with different doses of pheromone as single odour or in the mixture (i.e. 1 ng of pheromone for virgin and 10 ng for mated, see above) and therefore data were not comparable.

Statistical assumptions of homogeneity of variance (Levene's test, Box M), normality and sphericity (Mauchly's test) were checked. Violation of sphericity was overcome by using Greenhouse-Geisser correction for the degrees of freedom (df) if necessary, thus the df were not integer numbers.

For behavioural experiments, statistical differences (p<0.05) of responses to the different doses of heptanal were evaluated using a chi-square test.

## Supporting Information

Figure S1 **Responses of pheromone-responding ORNs stimulated with control stimuli.** Stimulation with pipettes containing one filter paper with pheromone elicited responses, which were not significantly different from responses to pipettes containing one filter paper with pheromone (1 ng/10 µl, phe) and a second clean filter paper (cfp) or a second filter paper with mineral oil (10 µl, oil). Stimulation with mineral oil (10 µl) alone did not induce any ORN response (n = 9 for each stimulus type). For statistical analysis see text. The box represents the interquartile range (IQR) of the data, the horizontal line inside the box represents the median. The whiskers show the range of the remaining sample.
(EPS)
